# The expanding Asgard archaea invoke novel insights into Tree of Life and eukaryogenesis

**DOI:** 10.1002/mlf2.12048

**Published:** 2022-12-18

**Authors:** Zhichao Zhou, Yang Liu, Karthik Anantharaman, Meng Li

**Affiliations:** ^1^ Department of Bacteriology University of Wisconsin–Madison Madison Wisconsin USA; ^2^ Archaeal Biology Center, Institute for Advanced Study Shenzhen University Shenzhen China; ^3^ Shenzhen Key Laboratory of Marine Microbiome Engineering, Institute for Advanced Study Shenzhen University Shenzhen China

**Keywords:** archaea, Carl Woese, eukaryogenesis, Tree of Life

## Abstract

The division of organisms on the Tree of Life into either a three‐domain (3D) tree or a two‐domain (2D) tree has been disputed for a long time. Ever since the discovery of Archaea by Carl Woese in 1977 using 16S ribosomal RNA sequence as the evolutionary marker, there has been a great advance in our knowledge of not only the growing diversity of Archaea but also the evolutionary relationships between different lineages of living organisms. Here, we present this perspective to summarize the progress of archaeal diversity and changing notion of the Tree of Life. Meanwhile, we provide the latest progress in genomics/physiology‐based discovery of Asgard archaeal lineages as the closest relative of Eukaryotes. Furthermore, we propose three major directions for future research on exploring the “next one” closest Eukaryote relative, deciphering the function of archaeal eukaryotic signature proteins and eukaryogenesis from both genomic and physiological aspects, and understanding the roles of horizontal gene transfer, viruses, and mobile elements in eukaryogenesis.

## THE DISCOVERY OF ARCHAEA AND TWO NOTIONS OF THE TREE OF LIFE

**Figure 1:**
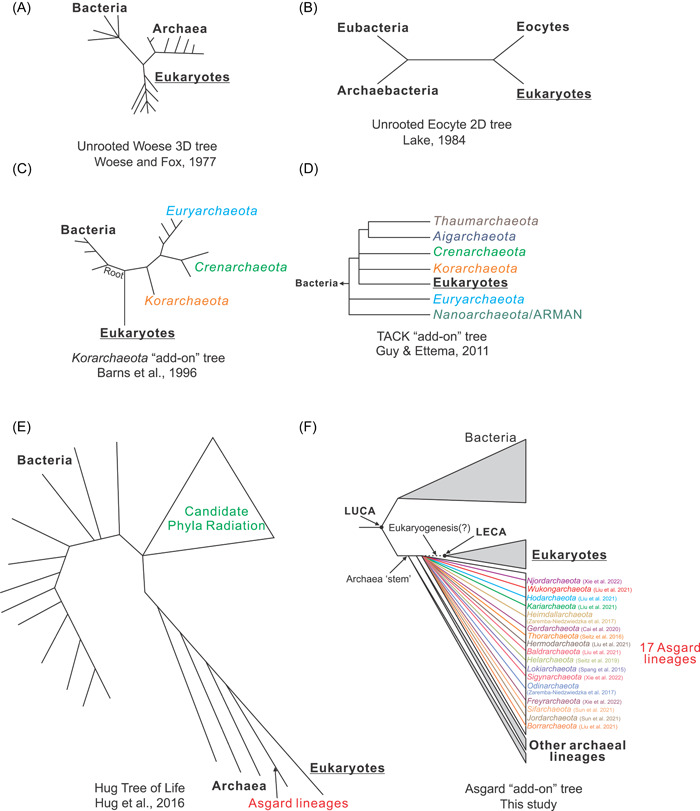
Progress in the “Tree of Life” as increasing archaeal lineages having been identified in recent decades. (A) The unrooted Woese 3D tree first conceived in 1977 by Carl Woese and Fox^1^. (B) The unrooted Eocyte 2D tree drawn in 1988 by James Lake^2^. (C) The *Korarchaeota* “add‐on” tree constructed in 1996 by Barnes et al.^3^, (D) The TACK “add‐on” tree constructed in 2011 by Guy and Ettema^4^. (E) The Tree of Life constructed in 2016 by Hug et al.^5^. (F) The Asgard “add‐on” tree constructed in 2022 in this work. This tree includes all the 17 Asgard lineages to have been discovered since 2015. The shape of this tree is derived from Figure 1 by Zhou et al.^6^. All these trees are conceptualized trees based on the original topology. The branching order of Asgard lineages within the Asgard “add‐on” tree was not represented for simplicity. ARMAN, Archaeal Richmond Mine Acidophilic Nanoorganisms; LECA, last eukaryote common ancestor; LUCA, last universal common ancestor.

The wide usage of 16S ribosomal RNA (rRNA) as the gold standard for determining the taxonomy of prokaryotes roots back to the late 1970s. In 1977, Woese and Fox discovered a new kind of microbial life that is not the same as typical bacteria based on 16S rRNA phylogeny^1^. They initially named them “archaebacteria”, representing a new group of life besides Bacteria and Eukarya. This is the emergence of the notion of a three‐domain (3D) Tree of Life represented by—Bacteria, Archaea, and Eukarya (Figure 1A). They proposed this name because they believed “archaebacteria” were ancient, living in extreme environments, and had robust evolutionary connections to the organisms living on early earth^7^. The short name “archaea” was then widely adopted by the scientific community after this finding was gradually accepted.

Nevertheless, in the late 1980s, there came two theories to deal with the placement of Archaea and Eukarya. One was the “Eocyte tree” model developed by James Lake^8^. Eocytes, a group of extreme thermophiles without a nucleus, are believed to be the ancestors of Eukaryotes. They formed the “Karyotes” branch with Eukaryotes, comparable to the other branch composed of Prokaryotes (Figure 1B)^2^, ^8^. On the contrary, Woese and Fox^1^ claimed the division of Prokaryotes into two life domains—Bacteria and Archaea, and posited the Eukaryote branch as an independent lineage (Figure 1A). Beyond this, other pieces of biochemical evidence were added; phylogenetic trees based on proteins encoding the information‐processing machinery (RNA polymerases, DNA‐dependent RNA polymerases, and ribosomal proteins) were observed to be congruent with the 3D tree^9^, ^10^, ^11^.

When it comes to the late 1990s and 2000s, the topology of 3D tree was depicted with the following features^12^: (1) the root of three domains could not be determined from rRNA sequences; (2) Eukarya and Archaea had a common ancestral branch, which was different from the Bacteria branch; (3) Eukarya and Archaea were close but were separated into two branches; in this manner, Archaea were distantly related to Bacteria, and Archaea and Bacteria were not monophyletic as previously thought^13^. The establishment of the 3D tree since the late 1970s soon replaced the old taxonomic clustering system mainly based on morphological subjects^13^. It challenged the simple division of Eukarya and Prokarya by the existence of a membrane‐bounded nucleus. In terms of the evolutionary line of cell nucleus genetic material, the Eukarya line is as ancient as that of Archaea in the ribosomal RNA‐based 3D tree (Figure 1A); in this way, Eukarya was posited not directly evolving from either Archaea or Bacteria.

## THE GROWING DIVERSITY OF ARCHAEA

Currently, based on NCBI Taxonomy data updated through October 2022, there are 39 phylum‐level archaeal lineages: *Euryarchaeota*, *Thermoplasmatota*, *Hydrothermarchaeota*, TACK (*Thaumarchaeota*, *Aigarchaeota*, *Crenarchaeota*, *Korarchaeota*) group (*n* = 10), DPANN (*Diapherotrites*, *Parvarchaeota*, *Aenigmarchaeota*, *Nanoarchaeota*, *Nanohaloarchaeota*) group (*n* = 11), and Asgard group (*n* = 15). The classification of Archaea started from quite a few numbers of lineages. Ever since the establishment of Archaea, *Euryarchaeota* and *Crenarchaeota* were the first two major clades identified in Archaea^14^. *Euryarchaeota* contains methanogens, anaerobic methanotrophs, and halophilic archaea^14^. It also includes thermophilic *Thermococcales*, which usually are acidophilic and do not contain a cell wall^14^. *Thermoplasmatota* was first proposed by Rinke et al.^15^ in 2019. It contains *Poseidoniia* (formerly recognized as Marine Group II and III), *Thermoplasmata*, and Deep‐sea Hydrothermal Vent Euryarchaeota 2 (DHVE2) group. *Hydrothermarchaeota* formerly recognized as the Marine Benthic Group E was proposed as a new archaea phylum by Carr et al.^16^ in 2019. It is named after its first discovered place of the continental slope and abyssal sediments^17^.

*Crenarchaeota* was the most abundant component within the TACK group. *Crenarchaeota* mostly comprises of hyperthermophiles that are discovered in geothermally‐heated/acidic hot spring environments^14^. Most of them are obligate anaerobes with either chemoorganotrophic or chemolithotrophic lifestyles^14^. Many of them also live in hot springs, submarine volcanic, or hydrothermal vent habitats^14^. In the middle 1990s, *Korarchaeota* was added to the Tree of Life (Figure 1C)^3^. *Korarchaeum cryptofilum* is the first representative of this phylum^18^. It is an obligate anaerobe with a chemoorganotrophic and hyperthermophilic lifestyle. From its genome, it is posited to live a fermentative lifestyle using peptides and amino acids.^14^ Additionally, it lacks the machinery to perform anaerobic respiration and likely depends on other community members in the form of a mutual dependence relationship to acquire co‐factors^14^. It was assumed that all archaeal lineages were obligate anaerobes and lived in extreme environments until the discovery of *Thaumarchaeota* in the 2000s. The first isolated strain of *Thaumarchaeota*, *Nitrosopumilus maritimus*, is a representative of their aerobic lifestyle dependent on oxidizing ammonia to nitrite^19^. It is adapted to a life that can grow at ammonia concentrations that are 100 times lower than those required by bacterial nitrifiers, which explains their widespread distribution in the open ocean^14^. *Aigarchaeota* formerly recognized as the Hot Water Crenarchaeotic Group I (HWCG I group) was proposed to be a new phylum by Nunoura et al.^20^ in 2010. It contains two candidate genera—*Caldiarchaeum* and *Calditenuis*. The study of the *Aigarchaeota* genome reveals the first evidence of eukaryotic ubiquitin system homologs in archaea^20^.

The DPANN superphylum of Archaea was first proposed in 2013^21^. The name is an acronym of the initials of the first five groups discovered—*Diapherotrites*, *Parvarchaeota*, *Aenigmarchaeota*, *Nanoarchaeota*, and *Nanohaloarchaeota*. Later on, many other groups were added—*Altiarchaeota*, *Huberarchaea*, *Micrarchaeota*, *Pacearchaeota*, *Undinarchaeota*, and *Woesearchaeota*. They are characterized as ultra‐small archaea with nanometric cell size. They have small genome sizes with limited metabolic capabilities. Many members of DPANN depend on symbiotic/parasitic interactions with other organisms^22^, ^23^. The specific placement of DPANN lineages on the archaeal tree is somewhat controversial^23^. Studies have claimed DPANN to be a deep‐branching archaeal superphylum in the basal position of the archaeal tree^24^, ^25^. In contrast, others have suggested that DPANN lineages are fast‐evolving species that have high mutational rates leading them to be placed at the base of the phylogenetic tree as results of long‐branch attraction artifacts^26^.

The last archaeal group is the newly discovered Asgard group which is believed to bridge the gap between Archaea and Eukarya. The emergence and development of Asgard group are described in detail in the following section. Many new lineages under the four above‐mentioned archaeal groups have been discovered and characterized by the metagenomics method as well as the single‐cell genomics, transcriptomics, proteomics, and enrichment culturing methods. For example, *Marsarchaeota* ^27^, *Brockarchaeota* ^28^, *Bathyarchaeota* ^29^, *Geothermarchaeota* ^30^, and *Verstraetearchaeota* ^31^ are newly discovered phyla within the TACK superphylum. *Hadesarchaea* ^32^, *Methanofastidiosa* ^33^, *Theionarchaea* ^34^, and Nanohaloarchaea^35^ are newly discovered classes within *Euryarchaeota*; *Proteinoplasmatales* ^36^ and *Thermoprofundales* ^37^ are newly discovered orders within *Euryarchaeota* and *Thermoplasmatota*. The large volume of newly emerging archaea as well as bacteria has significantly increased the diversity and physiology knowledge but requiring improved classification and nomenclature. Consequently, a new classification platform, genome taxonomy database (GTDB), arises to achieve a better assessment of the diversity of archaea as well as bacteria based on genomic materials^38^. Based on the GTDB database (07‐RS207), there are 18 phylum‐level archaeal lineages. The archaeal reference phylogenomic unrooted tree containing 3412 representative species was inferred from 53 conserved marker proteins and decorated with GTDB taxonomy (Figure S1), showing a scene of flourishing branches and leaves of expanded archaeal lineages. Meanwhile, their corresponding NCBI taxonomy information was also provided (Supporting Information: Data S1). GTDB uses a relative evolutionary divergence method to delineate high‐ranking microbial taxa, which normalizes the contents of each phylum by quantitative criteria based on genetic distance^38^. Currently, the scientific community has reach a consensus statement for creating consistent rules for the nomenclature of uncultivated taxa^39^, meanwhile, a tool named SeqCode using the genomic sequence of prokaryote as the nomenclatural type^40^ is also introduced to solve the issue. The diversity of archaea to discover as well as the tools and platforms for classifying and naming microbial diversity will continue in the foreseeable future.

## CHANGING OF THE TREE OF LIFE (3D TO 2D)

Since the very beginning of the discovery of Archaea, it has been a dispute to solve the phylogenetic position of Archaea and Eukarya. In the early stage before the 1990s, Woese's 3D tree and Lake's “Eocyte tree” model are the major competing notions. However, both trees are quite limited by the small number of archaeal genomes available at that time. For instance, *Korarchaeota* was the third archaeal clade introduced to the 3D tree until the middle 1990s^3^. *Korarchaeota* branched before the bifurcation of *Crenarchaeota* and *Euryarchaeota* and even formed a sister clade to Eukarya^3^. On the other hand, Eocyte, which was believed to be a new biological kingdom in the “Eocyte tree” model, was later proved to be a member in *Crenarchaeota* ^41^. In addition, at that time, Archaea was limitedly represented by methanogens and halobacteria. It appears that the separation between Eocyte and Archaea at that time was caused by the absence of the intermediate archaea. Both examples indicate that the discovery of new archaeal clades will reform our knowledge of the Tree of Life, which leads to a better understanding of the emergence of eukaryotes.

Along with the increasing diversity of Archaea discovered, the general idea of the placement of Eukaryotes in the Tree of Life and the relationship between protoeukaryotes and the Archaea “stem” in the tree has gradually changed. A key issue is where to place the protoeukaryotes or the last eukaryote common ancestor (LECA) in the phylogenetic tree. The idea that LECA is parallel to the branch that leads to extant archaeal lineages produces the 3D tree model. However, placing LECA as rooted from the Archaea “stem” gives the 2D tree model^6^. Ever since the 1990s, increasingly available genomic data of microorganisms and advanced evolutionary reconstruction methods have driven the revival of 2D tree—which is obviously different from the previous paradigm of dividing life forms simply into Eukaryotes and Prokaryotes^6^ (based on the presence/absence of nucleus), or Karyotes and Prokaryotes (the “Eocyte tree” model)^8^. By analyzing 26 universally conserved protein clusters, one of the first studies with genomic evidence from newly discovered archaea looked back to the 2D tree theory, and Eukarya was placed as the sister clade of the TACK superphylum^4^ (Figure 1D). This study suggests that eukaryotes have an archaeal parent that is most likely affiliated with the TACK superphylum and that archaeal gene sets found in eukaryotes were vertically inherited from the archaeal parent. It was once hypothesized that Eukarya was nested within the TACK superphylum (close to *Korarchaeota*; see Figure 1D topology) or emerging as a sister group adjacent to the TACK superphylum^4^, ^42^.

In 2015, the Asgard superphylum was discovered and characterized as containing the lineages bridging Eukarya and Archaea^43^, ^44^, ^45^, ^46^, ^47^, ^48^, ^49^, ^50^. Since the early time of placing *Thorarchaeota* and *Lokiarchaeota* as the closest lineages to Eukarya in a 2016 “Tree of Life” (Figure 1E), currently, there have been 17 Asgard lineages reported (Figure 1F). The reconstruction of the phylogeny of Asgard achaea, other archaeal lineages, Bacteria, and Eukarya based on concatenated 29 universal markers supports either the origin of Eukarya within Asgard superphylum or a deeper branch of eukaryotic ancestor within Archaea—both suggest a 2D tree topology for the Tree of Life^45^. Simultaneously, Asgard archaea have been identified to contain a more expanded repertoire of eukaryotic signature proteins (ESPs) compared to the other archaeal lineages such as the TACK superphylum. The majority of Asgard ESPs belong to the “intracellular trafficking, secretion and vesicular transport” and “posttranslational modification, protein turnover and chaperones” functional categories^45^. Many of them are from the roadblock superfamily and small GTPases family—two families that constitute or build the intracellular membrane‐bound compartments of eukaryotes^45^, ^51^. Both the phylogeny and ESP evidence support the archaeal origin of eukaryotes in that protoeukaryotes are either rooted within Asgard or derived from an even basal branch within the archaea domain, thus supporting the topology of a 2D tree of life.

## FUTURE DIRECTIONS

### Unraveling the phylogenetic relationship between Archaea and Eukarya and identifying the closest relative of eukaryotes

Phylogenetic advances are needed to determine the placement between Archaea and Eukarya. The key issue is the usage of the marker set of proteins for these analyses. Normally, the disparity of different interphylum relationships is rooted in the choice of marker set. By ranking each marker based on the congruency of supporting the monophyly of well‐established archaeal lineages, Dombrowski et al.^52^ provided two sets of markers with reasonable robustness. Meanwhile, the metrics for tree certainty have been developed for identifying the set of single‐copy markers that perform best for predicting phylogeny^53^. Collectively, a reasonable approach to reconciling the distinction of different tree topologies would involve choosing a reliable marker set. Inclusion of conserved core genes with few or no signal of duplications and horizontal gene transfers (HGTs) either within or among genomes/lineages would be a possible solution. It is also suggested to use a set of custom‐ranked markers that are suitable to solve the depth of phylogeny^54^. Meanwhile, improved/balanced sampling of taxa for better representation of tree topology and lineage placement is required^53^.

Asgard superphylum has been discovered and characterized as the lineages bridging between Eukarya and Archaea^43^, ^44^, ^45^, ^46^, ^47^, ^48^, ^49^, ^50^. Ever since the discovery of the first Asgard archaea lineage of *Lokiarchaeota* in 2015^43^, the knowledge of the closest relative of eukaryotes continues to push forward. In 2017, Zaremba‐Niedzwiedzka et al.^44^ expanded Asgard superphylum with additional *Thor*‐, *Odin*‐, and *Heimdallarchaeota* and further suggested that Asgard archaea were affiliated with eukaryotes in phylogenomic analyses. Later on, many Asgard lineages were discovered in the recent 5 years from groups around the world^47^, ^48^, ^50^, ^55^. In 2021, *Wukongarchaeota*—the Asgard archaea named after Sun Wukong (The Monkey King), a figure in Chinese mythology—was discovered and currently believed to be the closest relative^45^, ^48^, ^54^. Based on different ways to reconstruct the phylogeny of eukaryotes and archaea, there is still some support for placing eukaryotes basal within archaea other than within the “expanded *Heimdallarchaeota*–*Wukongarchaeota*” branch^45^. Discovery of more close relatives of eukaryotes from diverse environments continues to take place today. The search for the “next relative” is not only an attempt to solve or refine the phylogeny puzzle, but also provides genomic context that is valuable to explore eukaryotic features of archaea in the context of the landmark of eukaryogenesis, deduce the metabolism of Asgard archaea ancestors, and the syntrophic relationship between protoeukaryotes and hosts^45^.

### The function of archaeal ESPs and eukaryogenesis

**Figure 2:**
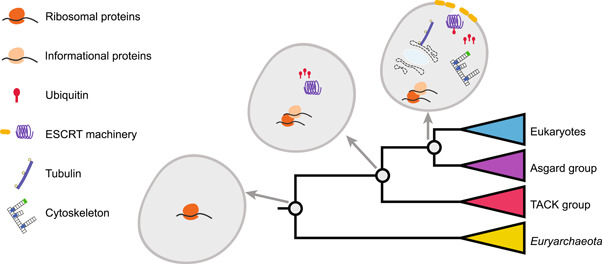
Eukaryotic signature proteins (ESPs) present in major archaeal groups. The emergence of homologs of ESPs along the schematic tree of Archaea (referred to Eme et al.^57^) is shown. The dash‐lined illustration of nuclei and endoplasmic reticulum currently has not been proven to be present in the common ancestor of eukaryotes and Asgard archaea. ESCRT, endosomal sorting complex required for transport.

In 2008, it was reported that the cell division machinery (Cdv) of *Crenarchaeota* has sequence homology to core domains with eukaryotic endosomal sorting complexes required for transport (ESCRT) machinery, which suggested common evolutionary origins between Archaea and Eukarya^56^. Later, more reports on ESPs and evidence of phylogenetic reconstruction indicated the close relationship between Archaea and Eukarya. When Asgard archaea were first uncovered with four phyla (*Loki*‐, *Thor*‐, *Odin*‐, and *Heimdallarchaeota*), their genomes encoded the highest number of ESPs among prokaryotes^43^, ^44^ (Figure 2). The functions of these ESPs mainly discovered in Asgard archaea can be categorized into trafficking machinery, cytoskeleton, ubiquitin system, and so forth^43^, ^44^, ^57^.

In 2021, more phylum‐level lineages of Asgard archaea were proposed including *Candidatus* *Wukongarchaeota* ^45^. Meanwhile, the increased number of Asgard genomes has resulted in a major expansion of the set of ESPs^45^, more than half of them have not been described previously^44^. The ESCRT machinery as components of the ubiquitin system has one of the most conserved genomic neighborhoods in the genomes of Asgard archaea, implying signatures of vertical inheritance of ESCRT machinery from the ancestor^45^. The physiological functions of the ESCRT genes in Asgard archaea have been studied^58^, ^59^, ^60^, ^61^. Collectively, this posits that these cell structure and trafficking machinery involving ESPs are functionally active in Asgard archaea, resembling activities in eukaryotic cells. However, phyletic patterns of ESPs in Asgard archaea are extremely patchy and each largely follows a lineage‐specific pattern, indicating that some ESPs might be acquired through HGT from a protoeukaryote to Asgard archaea^45^.

On the other hand, understanding the functions and evolutionary mechanisms of these ESPs in Asgard archaea can further support the bridging role of Asgard archaea in prokaryotes and eukaryotes. By comparing the enzymatic activity and selectivity from different life domains, scientists can study the ESPs in the context of evolution, such as a recent study on adenylate kinase^62^. Furthermore, using heterogeneous in vivo expression, recent studies have also demonstrated the functions of Asgard ESCRT‐III and Vsp4, implying that they have similar functional potential as their eukaryotic counterparts^61^, yet the regulating mechanism of Asgard ESCRT and its role on eukaryogenesis need further investigations. Meanwhile, as Asgard archaea possess eukaryotic‐like actin‐regulating proteins but with much more primitive characters, many actin regulators might be annotated with unknown functions and have different protein architectures as eukaryotes^63^. Since a sophisticated regulated actin cytoskeleton is the hallmark of eukaryotic cells, in the future, more efforts can be paid to determine and characterize other actin regulators and to link actin cytoskeleton to specific functions and structures in model Asgard archaea^63^.

### The roles of HGT, viruses, and mobile elements in eukaryogenesis

Based on inference from Asgard archaea genome collections, the deduced last Asgard archaea common ancestor (LACA) can use organic matter, that is, fatty acids, hydrocarbons, and aromatics^64^. HGT signals can be found in enzymes that are essential for these metabolisms beyond Asgard archaeal lineages and span across various groups in Bacteria and Archaea^64^. Additionally, it is believed that HGT contributes significantly to the symbiogenesis process between the archaeal progenitor and proto‐mitochondria^64^. Gene repertoire from free‐living bacterial cells occupies a considerable size within the eukaryotic cells by HGTs during the evolution process^65^. It remains unclear how these HGTs took place, thus the next step could be to investigate explicit HGT processes by identifying the origin of HGT donors along with the evolutionary process. In hypothesized eukaryogenesis models^45^, Asgard archaea interacted with bacterial partners in either different partnerships or forms of energy exchange. The direct or indirect HGTs of bacterial genetic components to protoeukaryotes via Asgard archaea may be possible, resulting in a high functional volume and flexibility for protoeukaryotes to customize their functional capacity and adapt to the environment. The discovery of HGT content, origin, and timeline will help reconstruct the scenario of the partnership during the ancient eukaryogenesis process.

Three Asgard archaea virus families were discovered to be widely distributed in different marine sediments—*Wyrdviruses*, *Verdandiviruses*, and *Skuldviruses* ^66^. *Verdandiviruses* and *Skuldviruses* belong to Realm *Duplodnaviria* and *Varidnaviria*, respectively. Although members from both *Duplodnaviria* and *Varidnaviria* can also infect eukaryotes, and have been proposed present in LACA and the last universal cellular ancestor^66^, current results indicate no direct evidence of these two Asgard archaea virus families infecting eukaryotes. Simultaneously, two other studies also reported Asgard archaea viruses^67^, ^68^. They reported more eukaryotic virus features found in Asgard archaea viruses, that is, ~1%–5% genes associated with eukaryotic nucleocytoplasmic large DNA viruses and being able to hijack host ubiquitin systems. These newly discovered unique archaeal and eukaryotic virus hybridized features for Asgard archaea viruses consistently reflect the evolutionary position of their hosts. More research targeting viruses is encouraged to complement current findings due to little/no overlap of the currently discovered Asgard archaeal virome pools and their globally ubiquitous distribution. As Asgard archaea viruses are evolving along with the evolution of Asgard hosts, it remains unknown whether viruses have played promotive roles in the evolution of Asgard archaea or in eukaryogenesis. Furthermore, it is also intriguing to find whether there are any extant eukaryote viruses directly evolving from Asgard archaea viruses.

Viruses and mobile genetic elements (without viral feature proteins) constitute the mobilomes of Asgard archaea^66^, ^69^. They can still reflect remnant HGT signals that descend from ancient Asgard archaea during eukaryogenesis to extant Asgard archaeal genomes^69^. Expanded mobilomes in the future will help us better trace the evolutionary history of HGT events before or after eukaryogenesis. According to the conceptualized framework of the “Heimdall nucleation–decentralized innovation–hierarchical import” (HDH) model for explaining eukaryogenesis^69^, the step of forming fully fledged protoeukaryotes involved domain‐specific hierarchical HGTs indirectly through Asgard archaea lineages and/or other related lineages (such as TACK superphylum or other transitionary lineages between Asgard and TACK). The expanded genomic contents of Asgard and related archaea lineages will facilitate our understanding of eukaryotic complexity formation from its archaeal ancestor. Different Asgard archaeal lineages have considerably small overlaps on ESP pools, and their ESP pool volume seems to be not associated with their phylogenetic distance to eukaryotes. It is intriguing to examine why there is a high mobility of ESPs and what factors govern the distribution of ESPs among the Asgard archaeal lineages. Furthermore, since during evolutionary history, protoeukaryotes gained their functional complexity significantly through HGT and mobilomes from donors, it is unknown how the cells determine the fates of imported genes and orchestrate functional patches into essential, organized functions. Many aspects of this conceptualized framework require attention in the future. The newly discovered genomic and physiological content and proposed hypotheses will help us better understand eukaryogenesis.

## Supporting information


**Figure S1**.

Supporting information.
