# Supplementary material for: The expanding Asgard archaea invoke novel insights into Tree of Life and eukaryogenesis
Source: mLife. 2022 Dec 18;1(4):374–81. doi: 10.1002/mlf2.12048 (PMC10989744; doi:10.1002/mlf2.12048)
Supplement: Supplementary file 1 — Figure S1. [file MLF2-1-374-s002.pdf]

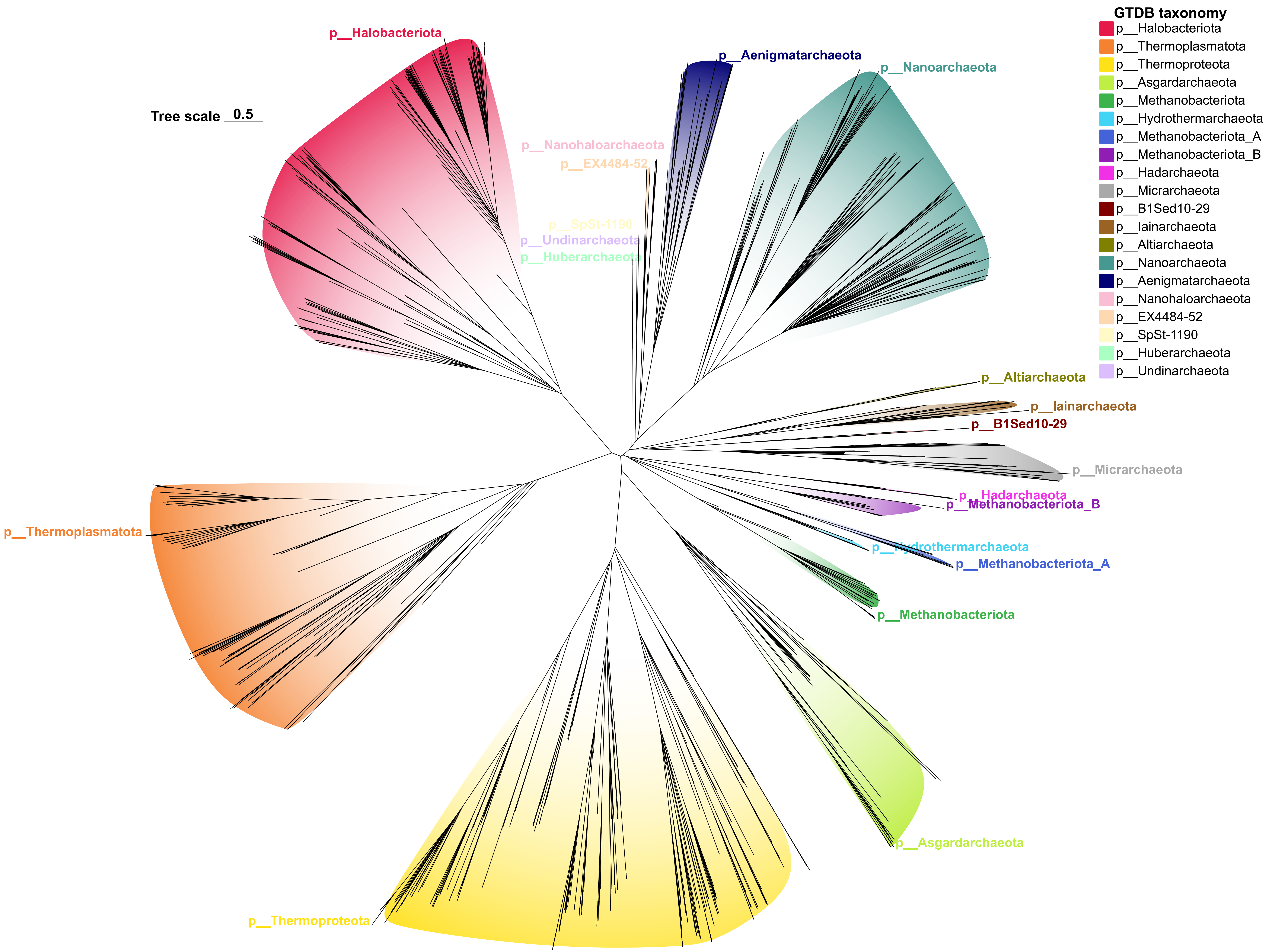

Tree scale 0.5
GTDB taxonomy
p__Halobacteriota
p__Thermoplasmatota
p__Thermoproteota
p__Asgardarchaeota
p__Methanobacteriota
p__Hydrothermarchaeota
p__Methanobacteriota_A
p__Methanobacteriota_B
p__Hadarchaeota
p__Micrarchaeota
p__B1Sed10-29
p__Iainarchaeota
p__Altiarchaeota
p__Nanoarchaeota
p__Aenigmatarchaeota
p__Nanohaloarchaeota
p__EX4484-52
p__SpSt-1190
p__Huberarchaeota
p__Undinarchaeota
